# Regional siderosis: a new challenge for iron chelation therapy

**DOI:** 10.3389/fphar.2013.00167

**Published:** 2013-12-31

**Authors:** Zvi Ioav Cabantchik, Arnold Munnich, Moussa B. Youdim, David Devos

**Affiliations:** ^1^Department of Biological Chemistry, Adelina and Massimo Della Pergola Chair, Alexander Silberman Institute of Life Sciences, Hebrew University of JerusalemGivat Ram, Jerusalem, Israel; ^2^Clinical Research Unit, Medical Genetic Clinic and Research Unit INSERM 781, Hôpital Necker-Enfants Malades and Université Paris V René DescartesParis, France; ^3^Technion-Rappaport Family Faculty of Medicine, Eve Topf Center of ExcellenceHaifa, Israel; ^4^Department of Medical Pharmacology, EA1046, Faculty of Medicine, Lille Nord de France University and Lille University Medical CenterLille, France

**Keywords:** iron, chelators, sideroblastic anemia, neurodegeneration, Parkinson's disease, Friedereich ataxia

## Abstract

The traditional role of iron chelation therapy has been to reduce body iron burden via chelation of excess metal from organs and fluids and its excretion via biliary-fecal and/or urinary routes. In their present use for hemosiderosis, chelation regimens might not be suitable for treating disorders of iron maldistribution, as those are characterized by toxic islands of siderosis appearing in a background of normal or subnormal iron levels (e.g., sideroblastic anemias, neuro- and cardio-siderosis in Friedreich ataxia- and neurosiderosis in Parkinson's disease). We aimed at clearing local siderosis from aberrant labile metal that promotes oxidative damage, without interfering with essential local functions or with hematological iron-associated properties. For this purpose we introduced a conservative mode of iron chelation of dual activity, one based on scavenging labile metal but also redeploying it to cell acceptors or to physiological transferrin. The “scavenging and redeployment” mode of action was designed both for correcting aberrant iron distribution and also for minimizing/preventing systemic loss of chelated metal. We first examine cell models that recapitulate iron maldistribution and associated dysfunctions identified with Friedreich ataxia and Parkinson's disease and use them to explore the ability of the double-acting agent deferiprone, an orally active chelator, to mediate iron scavenging and redeployment and thereby causing functional improvement. We subsequently evaluate the concept in translational models of disease and finally assess its therapeutic potential in prospective double-blind pilot clinical trials. We claim that any chelator applied to diseases of regional siderosis, cardiac, neuronal or endocrine ought to preserve both systemic and regional iron levels. The proposed deferiprone-based therapy has provided a paradigm for treating regional types of siderosis without affecting hematological parameters and systemic functions.

## Introduction

Iron homeostasis relies on the orchestration of a network of systemic and cellular mechanisms for the acquisition, internal distribution and use of iron (Cairo and Recalcati, 2007; Camaschella, 2009; Fleming and Ponka, 2012). Disruption of links in the metabolic network can lead to excessive iron accumulation in particular cell compartments or tissues (causing localized siderosis and thus damage) and also to impaired reuse of iron, generating a “deficiency in the midst of abundance” or vice versa (Breuer and Cabantchik, 2009; Rouault, 2012, 2013). This impairment translates into acquired or inherited anaemias (Camaschella, 2009; Fleming and Ponka, 2012; Rouault, 2012)and other disorders that display regional siderosis in organs such as heart, (Wood, 2007; Pennell et al., 2011; Fleming and Ponka, 2012; Rouault, 2012) brain (Berg et al., 2002; Zecca et al., 2004; Benarroch, 2009; Li et al., 2011; Sian-Hulsmann et al., 2011; Rouault, 2013)and other tissues(Pietrangelo, 2007). In all these disorders, the simultaneous dearth and surplus of iron pose new challenges in drug therapy, with the need to (i) detoxify discrete siderotic foci without affecting essential iron-dependent functions elsewhere and, conversely, (ii) replenish iron-deprived regions without further overloading those already in surplus. Ultimately, addressing the upstream factors that lead to iron maldistribution might lead to more effective therapies than those attempting to correct end-organ dysfunctions. (Camaschella, 2013) However, current tools for clinical intervention are limited to agents designed to treat systemic siderosis (Berdoukas et al., 2011; Ma et al., 2012; Camaschella, 2013) or iron deficiency (Weiss and Goodnough, 2005) but not discrete islands of siderosis that appear in a background of normal or subnormal iron levels (Pietrangelo, 2007; Breuer and Cabantchik, 2009).

We describe here a novel chelation concept designed to scavenge excess iron from regional foci of siderosis but also render the chelated metal metabolically reusable by redeploying it to cellular machineries or systemic acceptors such as transferrin.

## Iron chelation for the treatment of systemic siderosis

### Causes of tissue siderosis and treatment

**Figure 1:**
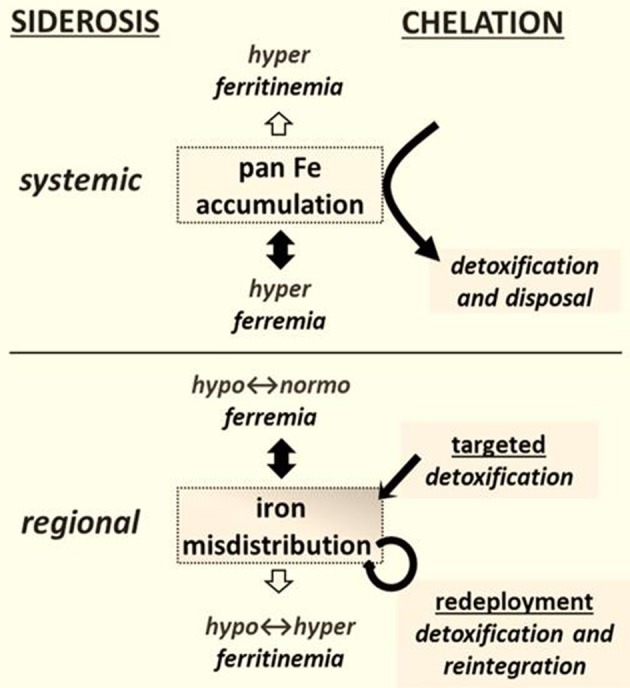
**Chelation modalities as treatments for siderosis.** Systemic siderosis (e.g., hemosiderosis) is characterized by elevated plasma iron levels (hyperferremia) that leads to organ iron accumulation and is generally characterized by elevated plasma ferritin (i.e., hyperferritinemia) that other than in a context of inflammation reflects iron stores. Chelation is designed to detoxify organs from surplus iron and dispose the latter via biliary or urinary secretion. Regional siderosis covers a wide spectrum of inherited disorders (e.g., Friedreich ataxia, sideroblastic anemias, iron-refractory-iron-deficiency anemia-IRIDA), and acquired disorders (e.g., anemia of chronic disease-ACD or cancer) that are characterized by a maldistribution of iron within cells of particular organs (e.g., cardiomyocytes or neurons or blast cells) or at the level of the organism (e.g., liver and spleen versus plasma). The plasma iron levels can span from subnormal (as in functional iron deficient anemias) to supranormal (as in sideroblastic anemias) and likewise those of plasma (or serum) ferritin levels. Chelation is designed here not merely to detoxify a siderotic region but where applicable render the chelated iron available for reuse. The new chelation modalities comprise (i) targeted detoxification, whereby a prochelator is activated at the target site by specific resident activators, as found in some brain areas (Sohn et al., 2008; Zheng et al., 2009) and (ii) iron redeployment, whereby a chelator that detoxifies cells from surplus iron and/or also scavenges essential iron reintegrates the metal into the erythron or specific tissues (Breuer and Cabantchik, 2009; Sohn et al., 2011).

This branch of pharmacology and medicinal chemistry has traditionally focused on the design, synthesis and application of drugs for relieving multi-organ iron accumulation (Fleming and Ponka, 2012) - particularly in patients with primary or secondary hemosiderosis (Figure 1) (Ma et al., 2012; Camaschella, 2013). In this context, excessive iron accumulation results from (i) enhanced erythrophagocytosis [which leads to increased iron deposition in hepatic and splenic reticuloendothelial system (RES) cells] (Weiss and Goodnough, 2005; Cairo and Recalcati, 2007; Fleming and Ponka, 2012) and/or (ii) the excessive release of iron into plasma by iron-rich RES cells or by an hyperabsorptive gut, resulting in high plasma iron levels to override transferrin's iron binding capacity (TIBC) and prompting the generation of non-physiological, non-transferrin-bound iron (NTBI) (Brissot et al., 2012; Cabantchik et al., 2013). Ultimately, chemically labile forms of plasma NTBI (referred to generically as labile plasma iron, LPI) infiltrate cells and attain therein toxic levels (Brissot et al., 2012; Cabantchik et al., 2013). (Comment: the term NTBI should be used with caution, as generically it is an apophasis-from the Greek απóϕασις-, namely something that is defined by what it is not).

In hemosiderosis, the toxicity that is associated with iron depends on a numbers of factors: (i) the chemical forms and concentrations of the NTBI/LPI, (ii) the LPI's membrane agencies and entry routes in particular cells; (iii) the cell's ability to handle “surplus” iron by sequestering it within “safe” ferritin sanctuaries and (iv) the cell's enzymatic and non-enzymatic means of counteracting the metal-catalyzed formation of reactive oxygen species (ROSs) involved in oxidative damage(Halliwell, 2006). *Although excessive iron ingress into cells can be deleterious, it is not the hyper-accumulated metal that is causatively associated with oxidative damage but rather a minor fraction referred to as labile cell iron (LCI)*(Halliwell, 2006; Cabantchik et al., 2013). The LCI is characterized by the: (i) redox activity, which largely determines not only its bio-catalytic roles but also its propensity to generate noxious ROSs from reactive oxygen intermediates of the respiratory chain and other oxygen-dependent reactions; (ii) ability to transfer the metal between natural ligands (including putative chaperons) and between compartments, which in turn determines the intracellular metal distribution, and (iii) the metal's amenability to chelation therapy via sequestration into a stable and potentially unreactive iron-chelates. Both LCI and LPI are direct pharmacological targets of chelators that act directly on plasma NTBI-LPI (Cabantchik et al., 2013) and/or permeate into cells and dissipate intracellular iron agglomerates (De Domenico et al., 2008).

In all forms of hemosiderosis, the therapeutic challenge is to attain a balance between iron detoxification and the maintenance of essential, iron-dependent functions. That requires the monitoring of indicators of systemic iron status in the plasma and of regional iron status in selected organs. Historically, (Pietrangelo, 2007; Berdoukas et al., 2011) the diagnosis and treatment of hemosiderosis were followed by (i) direct analysis of liver iron concentration in biopsies taken from what is the major iron storage organ and (ii) assays of serum levels of ferritin, which (in the absence of inflammation) is a surrogate (long-term) marker of liver-accumulated metal. Analytical methods for estimating urinary and fecal iron secretion have been applied in studies of body iron balance, with a view to directly assessing the efficacy of chelators in reducing the body's iron burden. (Berdoukas et al., 2011) The introduction of non-invasive methods for assessing tissue iron agglomerates was a major step forward in assessing long-term organ siderosis and the efficacy of iron chelation therapy. (Berdoukas et al., 2011) These assessments were initially performed in organs such as the liver [by using superconducting quantum interference device (SQUID) magnetometry] and, more recently, in the liver, heart and endocrine glands with T2 and T2^*^ nuclear magnetic resonance (NMR) spin relaxation methods (Wood, 2007; Berdoukas et al., 2011; Pennell et al., 2011; Camaschella, 2013). The most prominent example is the case of cardiac siderosis that results from transfusional hemosiderosis, in which the T2^*^ NMR relaxation time was validated (i) retrospectively for its correlation with cardiac iron measured in tissue biopsies and (ii) prospectively for its correlation with impaired cardiac function (Wood, 2007; Pennell et al., 2011).

Today's arsenal of iron chelators in clinical use comprises three agents approved for the treatment of transfusional hemosiderosis, (Berdoukas et al., 2011; Ma et al., 2012; Camaschella, 2013). The parenterally administered hexadentate deferrioxamine and the two orally active, bidentate deferiprone (DFP) and the tridentate deferasirox (DFX). These agents are used either alone or in various combinations designed to optimize the reduction of the body's iron burden by extracting excess iron from fluids and tissues and with time maintain NTBI-LPI at basal (i.e., sub-toxic) levels. (Berdoukas et al., 2011; Cabantchik et al., 2013).

## Iron chelation for the treatment of regional siderosis

### Causes and targets for treatment

Unlike hemosiderosis (in which plasma iron infiltrates cells in several different organs), regional siderosis mostly results from a disruption in cell homeostasis that causes iron to be abnormally distributed in certain cell types or specific organs (Fleming and Ponka, 2012; Rouault, 2012). In various types of sideroblastic anemia (Camaschella, 2009; Fleming and Ponka, 2012; Rouault, 2012) iron accumulates in mitochondria due to faulty use of the metal in the synthesis or secretion of iron sulfur clusters (ISCs) or heme (Rouault, 2012, 2013). A concomitant cytosolic iron depletion (Kakhlon et al., 2008; Rouault, 2012) generates a vicious cycle in which ever more incoming iron ends up in mitochondrial iron precipitates (Kakhlon et al., 2010; Whitnall et al., 2012). The damage caused by abnormal intracellular metal distribution has two major components: (i) toxic mitochondrial iron accumulation *per se* (mostly as labile/toxic iron-oxides) and (ii) a metabolic decline in cell iron-dependent activities (due to faulty synthesis of essential hemoproteins or ISC proteins) (Kakhlon et al., 2008; Rouault, 2012). Abnormal iron distribution is also observed in systemic functional iron deficiencies (Camaschella, 2009; Fleming and Ponka, 2012; Rouault, 2012). In acquired anemia of chronic disease (ACD) (Weiss and Goodnough, 2005) and in inherited iron-refractory-iron-deficiency-anemia (IRIDA), (Camaschella, 2009; Fleming and Ponka, 2012) macrophages of the RES system (in the spleen and liver) accumulate iron. The end results is plasma iron depletion and ensuing systemic iron deprivation. Moreover, in patients with chronic kidney disease and functional iron deficiency, intensive treatment with intravenous iron supplements can also cause the iatrogenic accumulation of macromolecular iron-sugar aggregates in spleen and liver. This can also be identified by T2^*^ NMR as iron overload (Ghoti et al., 2012). However, iron accumulation in the RES is *per se* not pathological, since apparently no labile iron is generated as long as the metal is safely shielded within shells of protein (e.g., ferritin) or saccharide (e.g., sucrose or dextran).

Iron maldistribution is also found in various neurodegenerative disorders(Zecca et al., 2004; Weinreb et al., 2010; Li et al., 2011; Pichler et al., 2013; Rouault, 2013).In the inherited movement disorder Friedreich's ataxia (FRDA), siderosis manifests itself first in the brain's dentate nuclei and ultimately in the heart (Rouault, 2013). In Parkinson's disease (PD), the dopaminergic neurons of the substantia nigra pars compacta progressively succumb to oxidative stress (Zecca et al., 2004; Halliwell, 2006; Li et al., 2011; Sian-Hulsmann et al., 2011; Pichler et al., 2013).

However, the contribution of brain siderosis to the etiology of PD is subject to debate—largely because opposing hypotheses have mostly relied on correlations between functional or biophysical indices of PD and indicators of systemic, rather than local and/or labile, iron levels (Zecca et al., 2004; Hider et al., 2011; Li et al., 2011) Those studies included trials of chelators that affect systemic iron but not necessarily brain iron, (Li et al., 2011; Mounsey and Teismann, 2012) or genetic factors that affect systemic iron levels [e.g., HFE and TMPRSS6 gene mutations (Camaschella, 2009, 2013; Pichler et al., 2013)] that in turn might be inversely correlated with PD severity (Pichler et al., 2013) and possibly with iron accumulated in the substantia nigra. Moreover, in regional siderosis (whether inherited or acquired), detection of iron agglomerates by T2^*^ MRI or histochemical staining is not necessarily indicative of an existing or impending disease state, just as failure to detect regional iron agglomerates does not rule out an existing or impending disease state.

By analogy with cardiac siderosis, we hypothesized that a causal relationship between iron and disease would be revealed if the course of disease is modified by selectively modifying labile iron levels in regions of siderotic damage (Breuer and Cabantchik, 2009; Kakhlon et al., 2010) We further reasoned that removal of the toxic labile component by “selective” chelation might suffice for relieving iron maldistribution disorders in which siderosis is the major pathological factor (i.e., by prevention and/or restoration of otherwise affected functions) (Figure 1). However, for disorders in which siderosis results from an inherited inability to use the metal for the production of essential ISCs or heme (as in sideroblastic anemia and FRDA), selective detoxification by targeted iron chelation (Zheng et al., 2009, 2010) might have only limited benefits. Moreover, in the absence of a safety mechanism that keeps chelated iron (resulting from systemic, non-specific chelation) in the body (Figure 1), the risk of iatrogenic iron deprivation might outweigh the benefits of regional detoxification (Breuer and Cabantchik, 2009). And conversely, the risk that systemic (parenteral) supply of polymeric iron supplements might pose to hypoferremic/ hyperritinemic patients that retain iron polymers their RES iron stores due to chronic inflammation for extended time periods (Ghoti et al., 2012) might outweigh modest changes in erythropoiesis evoked by increased transferrin saturation (Weiss and Goodnough, 2005). Prospective studies dealing with those issues are critically missing not only in clinical practice, but also in validated analysis of chemical stability of iron formulations as part of their shelf life and especially once delivered to patients via parenteral routes.

## Novel therapeutic strategies for regional siderosis

### Iron redeployment (figure 2)

This approach seeks to conserve regionally or systemically chelated iron by redeploying it to sites of use (i.e., to ISC or heme biosynthetic machineries or the physiological acceptor apotransferrin) (Sohn et al., 2008; Breuer and Cabantchik, 2009; Kakhlon et al., 2010). Redeployment (which is essentially applicable to any disorder of iron maldistribution, whether cellular or systemic) was initially assessed in cell-based models, (Kakhlon et al., 2008; Sohn et al., 2008, 2011; Moreau et al., 2013) corroborated in an animal model of regional siderosis (Moreau et al., 2013; Devos et al., 2013) and then translated to a clinical setting (Vreugdenhil et al., 1989; Boddaert et al., 2007; Wood, 2007; Abbruzzese et al., 2011; Kwiatkowski et al., 2012; Devos et al., 2013; Moreau et al., 2013). The membrane-permeant, bidentate DFP (1,2-dimethyl-3-hydroxy-pyridine-4-one) is the prototypic redeployment agent. It has a moderate ability to (i) rescue iron-overloaded cells by scavenging labile iron [especially from mitochondria, (Kakhlon et al., 2008, 2010; Devos et al., 2013; Moreau et al., 2013) the organelles most affected by cell iron accumulation and the ensuing labile-iron mediated oxidative damage (Halliwell, 2006)] and (ii) deliver chelated metal to extracellular apotransferrin (Sohn et al., 2008, 2011) (so that systemic iron losses are essentially avoided) and (iii) translocate across membranes (including the blood brain barrier) and gain access to foci of iron accumulation (thus conferring a degree of protection against metal-initiated oxidative damage) (Kakhlon et al., 2008; Sohn et al., 2011).

**Figure 2:**
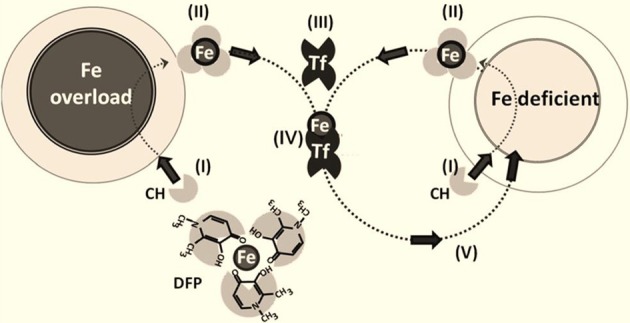
**Iron chelation and redeployment.** The redeployment strategy leans on permeant chelators (CH) **(I)** that gain access to cells (normal or Fe-overloaded), **(II)** chelate labile cell iron and, following egress from cells into plasma, transfer **(III)** the chelated Fe to circulating apotransferrin Tf to form TfFe **(IV)**, which in turn re-deploys the metal to Fe-deficient cells **(V)** in a conservative cycle. The various steps in the chelation-redeployment cycle have been demonstrated preclinically with the bidentate Fe **(III)** chelator deferiprone (DFP) (Sohn et al., 2011; Kakhlon et al., 2010; Fleming and Ponka, 2012). Given that DFP-Fe chelates donate metal to cell or plasma iron-acceptors (e.g., apo-Tf), the modus operandii of the chelators resembles siderophores or chaperons that redistribute iron across cells and their compartments.

The clinical assessment of DFP as a potential iron redeployment agent has been facilitated by (i) the drug's good safety profile in the treatment of hemosiderosis (Berdoukas et al., 2011) (ii) its proven chelating (and thus life-saving) effect in patients with severe cardiac siderosis (Wood, 2007; Berdoukas et al., 2011; Pennell et al., 2011) and (iii) the ability to support hemoglobin production in patients with ACD due to rheumatoid arthritis (Vreugdenhil et al., 1989). As a major goal was to redeploy iron and thus recycle the chelated metal, (Kakhlon et al., 2008; Breuer and Cabantchik, 2009) dose optimization required the siderotic region to be monitored for tangible indications of chelation and systemic iron-parameters (via the assessment of plasma transferrin saturation, complete blood counts and urinary iron) (Vreugdenhil et al., 1989; Boddaert et al., 2007; Wood, 2007; Abbruzzese et al., 2011; Kwiatkowski et al., 2012; Devos et al., 2013; Moreau et al., 2013).

The first attempts to assess the clinical safety and efficacy of up to 12 months of treatment with a moderate dose of DFP (20–30 mg/kg/d, initially combined with the antioxidant idebenone) were performed in adolescent patients with FRDA (Boddaert et al., 2007; Velasco-Sánchez et al., 2011). With the exception of a few cases of neutropenia (which resolved spontaneously after withdrawal of these patients from the study), DFP treatment modified neither plasma iron parameters nor the CBC but did reverse the cardiomyopathic hypertrophy (Velasco-Sánchez et al., 2011). The observed reduction in R2^*^ NMR values in the cerebellar dentate nuclei essentially proved that DFP probably acted in these brain areas by gaining direct access to LCI and (indirectly) dissipating large iron agglomerates (Boddaert et al., 2007). Similar hematological responses were observed following long-term DFP treatment of neurodegeneration with brain iron accumulation (NBIA) (Abbruzzese et al., 2011; Kwiatkowski et al., 2012) and, more recently, of PD patients on stable dopamine/dopaminergic regimens (Moreau et al., 2013) In the latter population, a 12 month oral DFP regimen was associated with clear reductions in the amount of agglomerated iron in the substantia nigra and attenuation of disease progression (according to the Unified Parkinson's Disease Rating Scale). The drug appeared to act by (i) cell iron detoxification and thereby a reduction in oxidative damage and (ii) reduction in enzymatic dopamine catabolism and/or non-enzymatic oxidation of naturally produced dopamine or administered dopamine agonists. At this stage we are not in a position to assess to what extent the protective effects afforded by chelation can be attributed solely to direct chelation of LCI and in turn reduction of metal-driven oxidations and ensuing cell damage. Chelation of labile iron at the level of prolyl-hydroxylases can lead to protective mechanisms involving HIF-dependent pathways, (Weinreb et al., 2010) particularly those leading to the local synthesis of erythropoietin, a hormone implicated in neuroprotection (Sturm et al., 2010; Matilla-Dueñas et al., 2013).

### Targeted (site-directed) chelation (figure 3)

**Figure 3:**
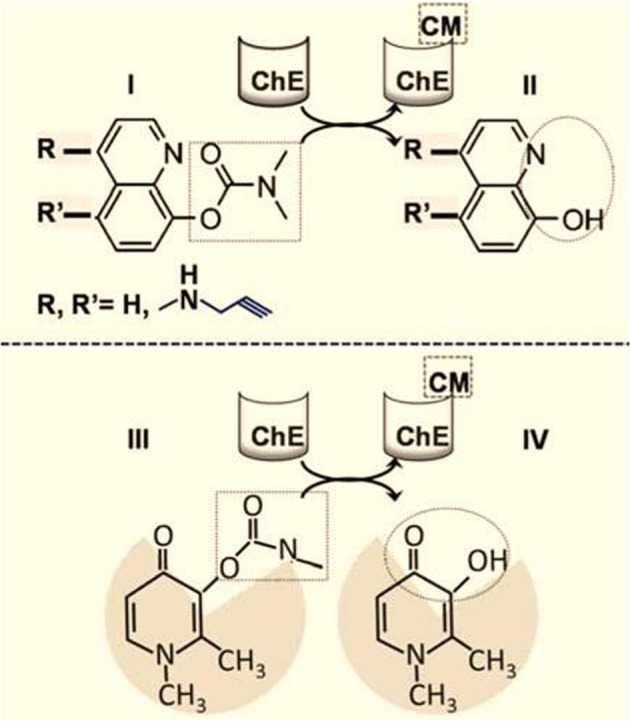
**Targeted brain iron detoxification via site-directed chelation.** The rationale is based on carbamate(CM)-blocked hydoxyquinoline (HQ) **(I)** susceptibility to cleavage by brain choline esterase (ChE) (Zheng et al., 2009; Sohn et al., 2011). The chelating unit **(II)** (depicted in the dotted circle) is released within areas of the brain with high ChE levels and is accompanied by concomitant carbamylation and inhibition of the ChE (CMChE). The HQ moiety can optionally be modified with R groups at the indicated positions; for example, a N-propargyl group can react with and inhibit monoamine oxidase (MAO), with the release of HQ-acid methylamine and hydrogen peroxide. **(III)** and **(IV)** represent the analogous schemes **(I)** and **(II)** as applied to the hydroxy pyridin-on deferiprone instead of HQ.

Targeted chelation has the inherent advantage of regional metal detoxification—particularly when it is based on a chelator that can be specifically activated *in situ* in the target region and can spare the organism from the risk of non-specific chelation and thus iron deprivation. At present, the best studied experimental approach is based on pro-chelators that are activated in the tissue, cell or organelle (i.e., at or close to the site of damage). This activation is achieved by an intrinsic (local) activation factor (e.g., an enzyme) that is only present or active in the target region (Sohn et al., 2008; Zheng et al., 2009) This concept is exemplified by the carbamylated hydroxyquinoline (HQ) pro-chelators that are converted by brain cholinesterases (ChEs) into active HQ chelators. As the ChE enzymes can be chemically modified and thereby inhibited by the leaving carbamate group (Figure 3), the approach has the potential benefit of addressing the dementia components of PD and Alzheimer's disease (AD). Moreover, the incorporation of N-propargylamine R groups confers to the HQ prochelators the additional capacity of monoamine oxidase (MAO) inhibition for the symptomatic treatment of PD (Sohn et al., 2008; Zheng et al., 2009). Several prochelators with these single- or double-agent features have been tested in animal models of neurodegenerative disorders (such as amyotrophic lateral sclerosis (ALS), AD and PD). They have both neuroprotective and neurorestorative effects (Sohn et al., 2008; Zheng et al., 2009; Weinreb et al., 2010) and do not appear to interfere (in the short term) with systemic iron and other HQ-chelatable biometals (such as copper and zinc). However, remaining to be assessed is to what extent the MAO-B versus MAO-A selectivity is retained over the long term treatment of chronic neurodegenerative condition.

## Conclusions and perspectives

Until recently, iron chelation therapy has focused primarily on the traditional role of reducing total iron burden that characterizes systemic siderosis (as in hemosiderosis) by chelating excess metal and excreting it via urinary and/or biliary-fecal routes (Berdoukas et al., 2011; Brissot et al., 2012; Ma et al., 2012; Camaschella, 2013). In the design of chelators, the major goal of systemic iron detoxification from excess iron, has hitherto mostly relied on high binding affinity properties and specificity of the chelating units for sequestering labile iron but also for generating non-toxic iron-chelates that ultimately should be disposed from the organism. The treatment of regional siderosis demanded a novel modality of chelation, one that can be used for clearing siderotic regions comprised of labile (toxic) metal but without interfering with essential local functions, thus sparing the patient's systemic iron pools. That selectivity property is of cardinal importance, since increased serum iron levels have recently been suggested by genome-wide meta-analysis (GWAS) to be correlated with a decreased risk of developing PD (Pichler et al., 2013), although, as we referred earlier, a direct role of the implicated genes (HFE and TMPRSS) in brain iron metabolism in general and on neurodegeneration in specific, still need to be properly evaluated, particularly in the context of PD.

The proposed chelation modalities designed for selective treatment of local siderosis have been tested in both cell-based and animal models. Those modalities are referred as: (i) **site-directed (targeted) chelation**, which relies on pro-chelating agents that are enzymatically converted into active chelators in various brain areas and act locally as metal-detoxifying agents (Sohn et al., 2008; Zheng et al., 2009) and (ii) **iron redeployment**, which aims at correcting foci of iron maldistribution by reutilizing tissue chelated metal and thus maintaining it within the body (Breuer and Cabantchik, 2009; Kakhlon et al., 2010).

Although several novel, brain-targeted pro-chelators have already displayed neuroprotective and neurorestorative properties in preclinical models of neurodegeneration, (Sohn et al., 2008; Zheng et al., 2009; Weinreb et al., 2010) their long-term clinical safety, specificity and efficacy have yet to be assessed. An iron redeployment agent that has already shown clinical benefits in the early stages of degenerative neurosiderosis is DFP, a moderately active, oral chelator with a proven record in the treatment of transfusional and hereditary cardiac siderosis (Wood, 2007; Berdoukas et al., 2011; Pennell et al., 2011). To date, most published placebo-controlled clinical studies have been carried out with DFP in combination with either idebenone (in FRDA) or with dopamine/dopaminergic drugs (in PD). Administration of the chelator was associated with significant benefits - possibly via disease-modifying mechanisms. Large, prospective, randomized clinical trials of DFP (alone and in combination with symptomatic drugs) are now needed, in order to properly appreciate the full therapeutic potential of new chelation modalities in regional forms of siderosis. This applies to not only neurosiderosis (as exemplified by FRDA, NBIA, and PD) but also various forms of hereditary and acquired sideroblastic anemia (Camaschella, 2009; Fleming and Ponka, 2012; Rouault, 2012) and other hallmark iron maldistribution disorders (Pietrangelo, 2007; Kakhlon et al., 2010; Rouault, 2012). The main challenge is to optimize existing treatments and develop novel specific, efficacious and safer drugs—possibly by combining a site-directed targeting component with a chelating moiety that is unmasked at the target sites and, like DFP, has metal-redeployment features (Figure 3).

## Author contributions

Writing of manuscript: a, first draft writing; b, review and critical comment; c, final draft writing. Zvi Ioav Cabantchik: a, b, c; Arnold Munnich: b; Moussa B. Youdim: c; David Devos: b,c.

### Conflict of interest statement

The authors have no financial disclosures to make or potential conflicts of interest to report in relation to this review. (The clinical trial on PD was funded by the French Ministry of Health PHRC grant Projet Hospitalier Recherche Clinique Protocol ID: 2008-006842-25), whereas Apopharma provided the compound deferiprone and instructions on its use. Zvi Ioav Cabantchik was supported by the A&M Della Pergola Chair in Life Sciences, served on the Scientific Advisory Board of Novartis ESB, received in the past research grants from NIH, EEC (5 and 6 framework), Israel Science Foundation, AFIRNE (Paris), Novartis, Shire and Apopharma (also lecture honoraria from the last two) and is presently a consultant for Aferrix, Ltd and Hinoman, Ltd, Israel. David Devos served on the Scientific Advisory Board for Novartis and Aguettant and has received PHRC grants from the French Ministry of Health and research funding from the ARSLA charity and honoraria from pharmaceutical companies for consultancy and lectures. Arnold Munnich is the head of the Pediatric Genetic Unit at Hospital Necker, Paris France. He has served as an advisor to Sanofi Genzyme until May 2013. He has presently no other appointment with commercial entities. Moussa B. Youdim receives Royalties from Teva Pharmaceutical Co. Israel and is Chief Scientific Officer co-owner and shareholder of Abital Pharma Pipeline Ltd, Israel, and of Avraham Pharmaceutical Co. Israel.
